# Quantitative analysis of light-induced ion segregation in mixed-halide perovskites

**DOI:** 10.1107/S1600576726002475

**Published:** 2026-05-20

**Authors:** Petr Machovec, Lukáš Horák, Milan Dopita, Neda Neykova, Lucie Landová, Jakub Holovský, Václav Holý

**Affiliations:** a Faculty of Mathematics and Physics Charles University Ke Karlovu 3 Prague Czechia; b Faculty of Electrical Engineering Czech Technical University in Prague Technická 2 Prague16627 Czechia; c Institute of Physics Czech Academy of Sciences v. v. i. Cukrovarnická 10 Prague16200 Czechia; d Faculty of Science Masaryk University Kotlářská 2 Brno61137 Czechia; Montanuniversität Leoben, Austria

**Keywords:** mixed-halide perovskites, light induced segregation, X-ray diffraction, strain, ion concentration distribution

## Abstract

Mixed-halide perovskites (MHPs) offer good band-gap tunability via stoichiometry changes, and such tunability is an essential property for the creation of multijunction solar cells. However, under illumination halide ions in MHPs segregate and create I- and Br-rich regions, which decrease the efficiency of potential solar cells. In this work, a method for a detailed investigation of the distribution of halide ions within an MHP during and after illumination is introduced. Calculations of the strain field created by the halide segregation were performed, and the obtained local displacement of atoms was used to calculate the X-ray diffuse scattering. By fitting the experimental data measured on a thin polycrystalline layer of FA_0.83_Cs_0.17_Pb(I_0.6_Br_0.4_)_3_ (where FA stands for formamidinium), the distribution of Br^−^ and I^−^ ions within an illuminated MHP was determined and the subsequent relaxation process of the segregation in the dark was tracked. The creation of highly Br-rich regions within a slightly I-rich volume during illumination was observed.

## Abstract 2

A quantitative X-ray diffraction approach is introduced to resolve light-induced halide segregation in mixed-halide perovskite thin films, revealing the formation of Br-rich regions and their slow incomplete relaxation in the dark.

## 1. Introduction

Metal halide perovskites have attracted significant attention in recent years due to their exceptional properties, such as long carrier lifetime (Dequilettes *et al.*, 2016 ▸; Ahmed *et al.*, 2018 ▸) and high photoluminescence quantum yields (Sutter-Fella *et al.*, 2016 ▸; Ahmed *et al.*, 2018 ▸; De Roo *et al.*, 2016 ▸; Kroupa *et al.*, 2018 ▸). These optoelectronic properties, combined with easy fabrication (Chen *et al.*, 2014 ▸; Jeon *et al.*, 2014 ▸; Mitzi *et al.*, 1963 ▸; Xiao *et al.*, 2014 ▸; Saidaminov *et al.*, 2015 ▸; Burschka *et al.*, 2013 ▸; Howard *et al.*, 2019 ▸), make metal halide perovskites promising candidates for applications in photovoltaics, LEDs and photodetectors.

The ideal halide perovskite structure is cubic *ABX*_3_, where *A* is a cation (methylammonium MA^+^, formamidinium FA^+^, Cs^+^), *B* is a metal cation (Pb^2+^, Sn^2+^*etc.*) and *X* is a halide anion (Cl^−^, Br^−^, I^−^). However, the ideal cubic structure can readily be distorted into a tetragonal or orthorhombic phase, with the transition driven by ionic size difference (Johnsson & Lemmens, 2008 ▸; Pradeep *et al.*, 2024 ▸).

The flexibility of the perovskite structure allows for the mixing of different ions on similar lattice sites. Such modifications offer relatively easy access to band-gap tunability, enhanced crystallinity and improved chemical stability. The wide band-gap range, extending from approximately 1 eV for lead iodide perovskites (Sa *et al.*, 2020 ▸; Noh *et al.*, 2013 ▸) to over 2 eV for lead bromide perovskites (Datta *et al.*, 2025 ▸; Noh *et al.*, 2013 ▸; Eperon *et al.*, 2014 ▸; Peter Amalathas *et al.*, 2025*a* ▸) and approaching 3 eV for lead chloride perovskites (Protesescu *et al.*, 2015 ▸; Lu *et al.*, 2025 ▸), makes perovskites ideal for both perovskite–perovskite and perov­skite–silicon tandem photovoltaics.

However, the long-term stability of mixed-halide perov­skites (MHPs) under illumination remains a critical challenge that limits practical deployment. One of the primary instability mechanisms is photoinduced halide ion segregation, where light exposure causes halide ions to migrate and form regions with different stoichiometries, leading to phase separation (Hoke *et al.*, 2015 ▸; Barker *et al.*, 2017 ▸). This results in a redshift of the band gap, altering the optical properties and reducing device efficiency over time. Before technologies based on MHPs can be commercially deployed, this instability needs to be addressed.

Numerous studies in recent years have tried to explain this instability. Models of structural rearrangement (Gottesman *et al.*, 2015 ▸), redistribution (Dequilettes *et al.*, 2016 ▸), decomposition (Juarez-Perez *et al.*, 2018 ▸; Li *et al.*, 2017 ▸; Tang *et al.*, 2016 ▸), grain boundaries (Ridzoňová *et al.*, 2022 ▸), trap states (Motti *et al.*, 2019 ▸), polarons (Bischak *et al.*, 2017 ▸, 2018 ▸) and band gaps (Pavlovetc *et al.*, 2021 ▸) have been proposed. Kerner *et al.* (2021 ▸) proposed a unifying model by identifying halide oxidation as the event initiating the demixing, but there is still disagreement across many experimental and theoretical studies on the mechanism and reversibility of segregation and subsequent remixing in MHPs. Furthermore, contradictory results have been reported on the timescale of the changes. While some studies report fast segregation and relaxation (Motti *et al.*, 2019 ▸; Nie *et al.*, 2016 ▸) with a characteristic time of less than a minute, other studies report characteristic times of tens of minutes or even hours (Kim *et al.*, 2018 ▸; Juarez-Perez *et al.*, 2018 ▸; Nie *et al.*, 2016 ▸; Gottesman *et al.*, 2015 ▸; Ridzoňová *et al.*, 2022 ▸).

In this work, we propose a method for the calculation of the influence of halide segregation in MHPs on the X-ray diffraction (XRD) peak profile. As a tool for the investigation of halide segregation, XRD has been used in many studies (Barker *et al.*, 2017 ▸; Hoke *et al.*, 2015 ▸; Sutter-Fella *et al.*, 2018 ▸; Knight *et al.*, 2021 ▸; Suchan *et al.*, 2023 ▸) but, to the best of our knowledge, no one has yet tried to analyse XRD patterns quantitatively to determine the distribution of the halide concentration in samples after illumination.

## 2. Computational method

For the simulation of deformation in a single crystal or in an individual grain of a polycrystal of an MHP, we assume that the deformation is caused solely by a random spatial fluctuation of the Br/I ratio, which can be characterized by the local Br concentration *c*_Br_(**r**). The deformation is purely elastic and, in the simulation, we neglect the relaxation of internal stresses at the sample surface, as well as plastic relaxation at grain boundaries, dislocations and other defects. For the XRD calculations, it is convenient to introduce a local displacement field **u**(**r**), defined with respect to an ideal structure corresponding to a homogeneous mean Br concentration. The procedure for calculating this displacement field is described in Appendix *A*[App appa]. Within the context of this work, the term ‘strain’ refers strictly to the deformation of this idealized homogeneous structure. It should not be confused with the stress-free strain, although the two quantities are interconvertible when the local Br concentration is known.

For the description of X-ray diffuse scattering from a single crystal of finite size, we use a standard model of a mosaic crystal, in which we assume that the crystal consists of randomly placed and randomly rotated mosaic blocks (Pietsch *et al.*, 2004 ▸). For simplicity, we assume that the mosaic blocks are spherical with random radii *R*; the radii obey the Gamma distribution with a mean value *R* ≡ *R*_0_ and root-mean-square (r.m.s.) deviation σ_*R*_ = 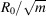, where *m* is the order of the Gamma distribution. The probability of finding two points **r**, **r**′ in the same block is
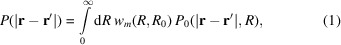
where 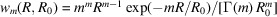 is the probability density of the Gamma distribution of order *m*. For *m* = 0 we can express *P* as (Holý *et al.*, 1993 ▸)
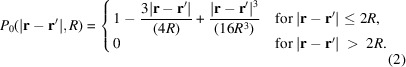


The random orientation of the blocks will be accounted for later. Since a powder-like curve is obtained from the calculated intensity by averaging over all orientations of the crystal lattice, the crystallographic orientation of the blocks has no influence on the final powder-like profile. Therefore, in our case the influence of mosaicity on the diffraction profile is only the broadening due to the finite size of the mosaic blocks.

Calculating diffuse X-ray scattering from an MHP crystal, we assume that (i) the kinematic approximation is valid, *i.e.* the blocks in the sample are much smaller than the X-ray extinction length (several micrometres), and (ii) the measured signal is averaged over all possible microstates [all block sizes and random functions of the Br concentration distribution *c*_Br_(**r**)]. The latter assumption is valid if the irradiated sample volume is much larger than *R* and ξ; ξ is the correlation length of the random function *c*_Br_(**r**).

The kinematic formula for the reciprocal-space distribution of the scattered intensity around a reciprocal-lattice point **h** reads (Pietsch *et al.*, 2004 ▸)
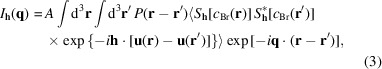
where *A* is a constant containing the intensity of the primary beam, the polarization factor and the Lorentz factor, among others; **q** ≡ Δ**Q** = **Q** − **h**, where **Q** = **K**_f_ − **K**_i_ is the diffraction vector and **K**_i_ and **K**_f_ are the wavevectors of the primary and scattered beams, respectively; and *S*_**h**_ is the structure factor of the MHP unit cell depending on the local Br concentration *c*_Br_(**r**),
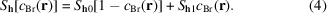
*S*_**h**0_ and *S*_**h**1_ are the structure factors of pure FA_0.83_Cs_0.17_PbI_3_ and FA_0.83_Cs_0.17_PbBr_3_ perovskites, respectively. The averaging 〈 〉 runs over all random functions *c*_Br_(**r**) and the reciprocal-lattice vector **h** is defined with respect to the MHP crystal structure with a mean Br concentration 〈*c*_Br_〉.

From a practical point of view, it is convenient to split the argument of the integral in (3) into diffuse and ‘coherent’ parts,
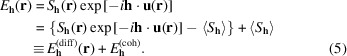
Then, after some manipulation, we obtain the following final formula, which is suitable for numerical simulations:
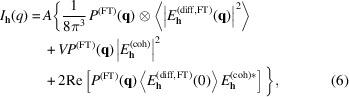
where *V* is the irradiated sample volume, ⊗ denotes convolution and superscript FT stands for Fourier transformation.

In each simulation, we generate *N* realizations of the random function *c*_Br_(**r**) assuming the correlation function in the form
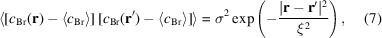
with r.m.s. deviation σ = 
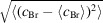
 and a given correlation length ξ. Additionally, we introduce an asymmetry of the random fluctuation, which enables us to modify the distribution of concentration. Specifically, it facilitates the creation of a concentration distribution characterized by small volumes of elevated concentration alongside larger volumes where the concentration is only marginally lower than the mean and vice versa. This asymmetry represents a situation in which bromine or iodine ions from a larger area are attracted to specific centres.

**Figure 1:**
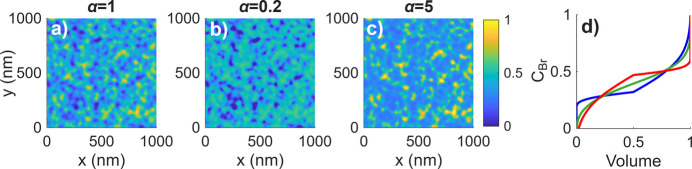
Examples of two-dimensional concentration profiles of Br generated with the correlation function in equation (7) for (*a*) α = 1, (*b*) α = 0.2 and (*c*) α = 5 with correlation length ξ = 30 nm and r.m.s. deviation σ = 0.15. The mean Br concentration for all distributions is 0.4. (*d*) Normalized cumulative histograms of the Br concentration, showing the fraction of the sample volume with Br concentration less than or equal to a given value, for α = 1 (green), α = 5 (blue) and α = 0.2 (red).

In the first step of a simulation run, the random fluctuation *c*_Br_(**r**) described by equation (7) is generated. Utilizing this concentration fluctuation *c*_Br_(**r**), a modified concentration 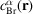 is constructed as follows:
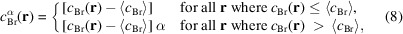
where the parameter α in the interval (0, ∞) is the parameter of asymmetry. The final asymmetrical fluctuation in bromine concentration, denoted 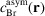, is then expressed as
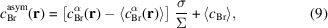
where σ is the original desired standard deviation and Σ is the standard deviation of 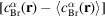. This ensures preservation of the correct mean value and standard deviation of the fluctuation. The parameter α acts as an amplitude-scaling factor for random fluctuations. Values α > 1 enhance positive deviations and suppress negative ones, leading to higher maxima and shallower minima. Conversely, values α < 1 attenuate positive deviations and deepen negative ones, resulting in lower maxima and deeper minima. To elucidate the role of α, Fig. 1 ▸ provides representative examples of the resulting distributions together with their cumulative histograms.

We calculate the final mean intensity as an average of the intensities calculated for different realizations of *c*_Br_(**r**),
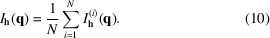


The number of configurations to average over, denoted *N*, must be selected such that the difference in the final intensity between *N* and *N* + 1 is negligible. It has been determined that *N* = 100 meets this criterion while concurrently being small enough to facilitate reasonable calculation times, and therefore it was used for all our calculations here.

To obtain a powder-like curve from the three-dimensional distribution of the intensity, we numerically integrate each function 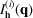 along a direction perpendicular to **h**, approximating the integration over a Debye sphere, and calculate the final intensity using equation (10).

**Figure 2:**
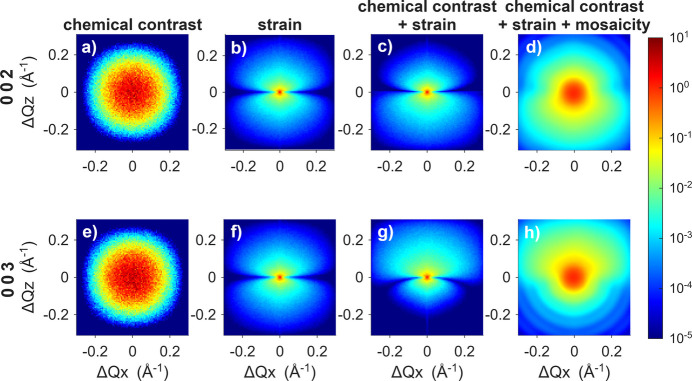
Simulated reciprocal-space maps of (*a*)–(*d*) 002 and (*e*)–(*f*) 003 diffraction maxima with (*a*) and (*e*) only chemical contrast included, (*b*) and (*f*) only strain included, (*c*) and (*g*) both chemical contrast and strain included, and (*d*) and (*h*) chemical contrast, strain and mosaicity included. The parameters of the random distribution of concentration are σ = 0.05, α = 1 and ξ = 1 nm, and the mosaic block size in (*d*) and (*h*) is *R* = 5 nm.

For the following considerations it is useful to express 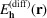 defined in equation (5) as
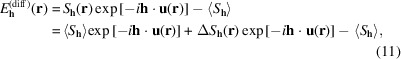
where 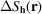 is the local deviation of the structure factor due to the local deviation in Br concentration. The two distinct contributions to 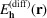 are the chemical contrast 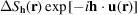 and the contribution from the fluctuating strain 〈*S*_**h**_〉{exp[−*i***h** · **u**(**r**)] − 1}. Here we separate these contributions only as a conceptual decomposition to explain the origin of the peak shape, whereas experimentally the measured intensity contains both inseparably. To illustrate the influence of the separate and combined contributions of chemical contrast, strain, both effects simultaneously and mosaicity, we show corresponding reciprocal-space maps (RSMs) simulated for random distributions of concentration with parameters σ = 0.05, α = 1 and ξ = 1 nm in Fig. 2 ▸.

**Figure 3:**
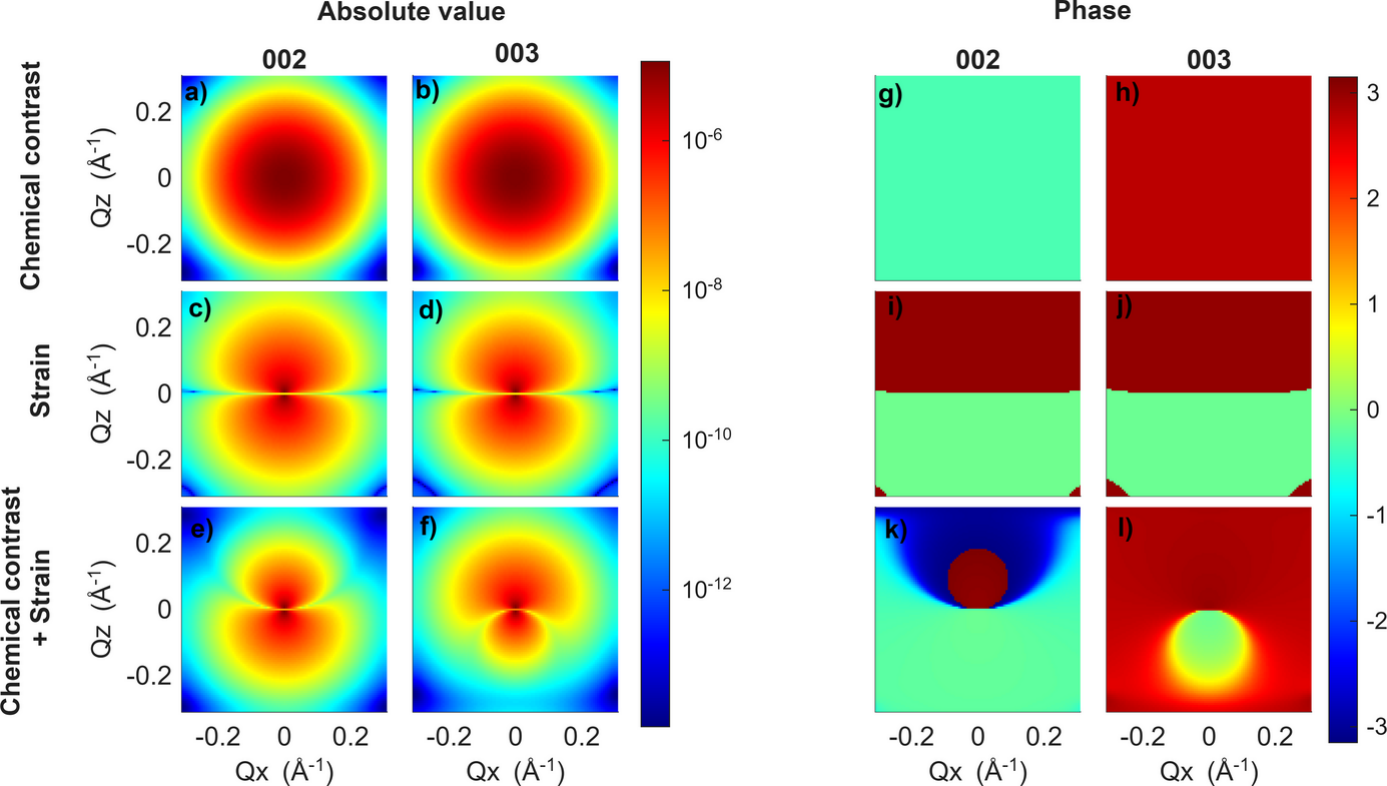
(*a*)–(*f*) Absolute values and (*g*)–(*l*) phases of simulated structure factors of a spherical inclusion of high Br concentration with a Gaussian profile. Panels (*a*), (*b*), (*g*) and (*h*) show the structure factor considering only chemical contrast, panels (*c*), (*d*), (*i*) and (*j*) show the structure factor considering only strain, and panels (*e*), (*f*), (*k*) and (*l*) show the structure factor considering both chemical contrast and strain.

While both chemical contrast and strain separately yield centrosymmetric intensity distributions, when both chemical contrast and strain are included together the resulting RSMs exhibit strong asymmetry. To understand the origin of this asymmetry, we will consider a simple distribution of *c*_Br_(**r**), specifically a spherical inclusion of either high or low Br concentration with a Gaussian profile of concentration. The parameters of this inclusion are a maximum deviation of concentration of 0.1 from the average concentration *c*_Br_ = 0.4 and a standard deviation of the Gaussian distribution of 10 nm. As shown in Fig. 3 ▸, the absolute values of the strain term create two symmetrical lobes but the phases of these lobes differ by π. Using equations (11) and (6) the symmetry of the lobes can be demonstrated for both a distribution of concentration with a spherical inclusion and a distribution of concentration with a correlation function described by equation (7). A full explanation is given in Appendix *B*[App appb]. Therefore, one lobe has the opposite phase to the other lobe and the signs of these phases depend on whether the inclusion has a high Br (tensile strain within the inclusion) or I (compressive strain within the inclusion) concentration. Absolute values of the Δ*S*_**h**_(**r**)exp[*i***h** · **u**(**r**)] term create a symmetrical spherical distribution but the phases of these spheres differ by approximately π for different diffraction maxima. As a result, when these two terms are added, the absolute values are added in one lobe and subtracted in the other, leading to an asymmetrical distribution of intensity. Therefore, the direction of this asymmetry is governed by the phase of the deviation of the structure factor Δ*S*_**h**_, which depends on the diffraction indices. A simple rule can be derived: for reflections with all Miller indices odd or all even, the asymmetry appears towards lower *Q*, whereas for mixed indices it shifts toward higher *Q*. This explanation is in many ways analogous to Huang scattering (Krivoglaz, 1996 ▸), where a contribution of the diffuse scattering from the deformed area around a defect core and the contribution of the defect core itself are superimposed. In Huang scattering the asymmetry has the same sign for all diffraction maxima and is determined simply by the sign of the strain.

High and low Br (low and high I) concentration inclusions create asymmetry in the same direction because the phases of both the strain and the chemical contrast flip signs for high versus low Br concentration. Therefore, as shown in Fig. 2 ▸, even for a random fluctuation that essentially consists of high and low Br concentration inclusions, we observe the described asymmetry.

**Figure 4:**
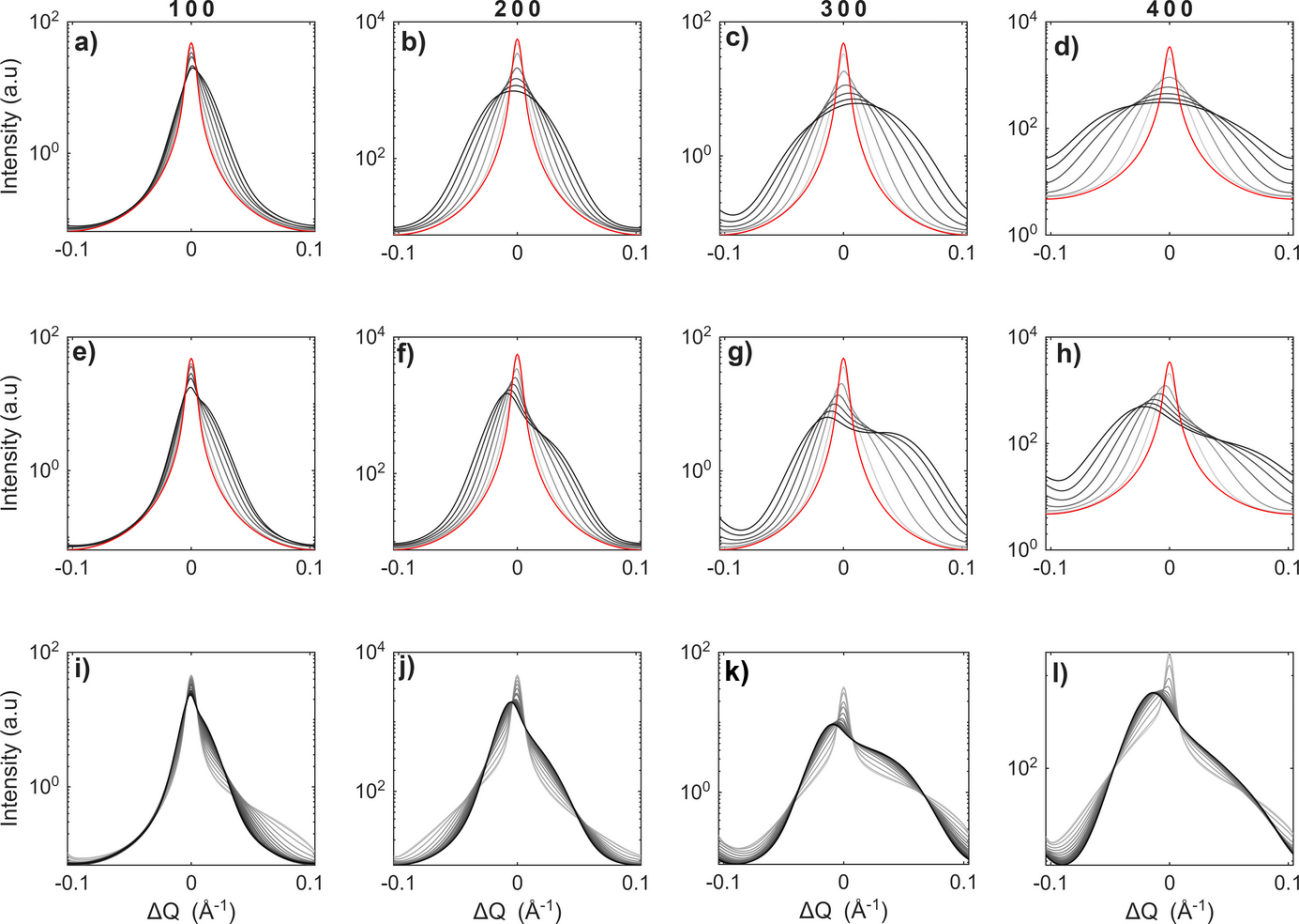
(*a*)–(*h*) Influence of r.m.s. deviation of the concentration on powder-like diffraction profiles. The red curves are profiles with uniform concentration. For the grey curves, values of σ go from 0.05 (light grey) to 0.3 (black). In plots (*a*)–(*d*) the random fluctuation of the concentration is symmetrical (α = 1) and in (*e*)–(*h*) the random fluctuation of the concentration prioritizes small regions with much higher Br concentration than the average concentration and large regions with slightly lower Br concentration than the average concentration (α = 5). Plots (*i*)–(*l*) show the influence of correlation length on the diffraction profile; the values go from ξ = 0.5 nm (light grey) to ξ = 17.5 nm (black) in 0.5 nm increments. All shown simulations are for a mosaic block size of 50 nm.

To demonstrate the effects of different parameters on simulated powder-like diffraction profiles and to evaluate the sensitivity of the model to individual parameters, a series of simulations were performed for various parameter values. The influence of different parameters is illustrated in Fig. 4 ▸. As expected, with increasing σ we observe broadening of the profiles accompanied by a decrease in the maximum intensity. The asymmetry of diffraction maxima described in the previous paragraph is visible for a symmetrical random fluctuation of the concentration (α = 1).

For an asymmetrical random fluctuation of the concentration (α ≠ 1) the asymmetry is mostly covered by the asymmetry of the concentration. This is demonstrated in Figs. 4 ▸(*e*)–4 ▸(*h*), where for α ≠ 1 we observe asymmetry of all diffraction maxima to the same side. For α < 1 the asymmetry is to lower *Q* (not shown here) and for α > 1 the asymmetry is to higher *Q*. The model is sensitive to the values of α in the interval (0.2, 5); outside of this interval the peak shape no longer changes with changing α. This asymmetry is accompanied by a decrease in maximum intensity and a slight shift in the position of the maximum intensity. This shift is understandable, because most of the volume now has a slightly higher or lower Br concentration and therefore slightly lower or higher lattice parameters.

The dependence of the profile shape on the correlation length is demonstrated in Figs. 4 ▸(*i*)–4 ▸(*l*). For correlation lengths under 7 nm a sharp peak is present, while for longer correlation lengths the profiles are broader and the central sharp peak disappears. Importantly, for correlation lengths above 15 nm, the shape of the peak is no longer influenced by changes in ξ.

It follows from our analysis that no particular *hkl* reflections are intrinsically more sensitive to specific parameters. Nevertheless, the dependence of peak broadening on the scattering-vector magnitude differs among parameters. Consequently, measuring and fitting as many reflections as possible is essential to enhance sensitivity and reduce parameter correlations. For example, reliable determination of α requires reflections with both equal-parity and mixed-parity Miller indices.

## 3. Experimental

### 3.1. Sample preparation

To demonstrate this method, thin films with chemical composition FA_0.83_Cs_0.17_Pb(I_0.6_Br_0.4_)_3_ were prepared on glass and silicon substrates using a one-step antisolvent approach. The precursor solution was obtained by dissolving the cation sources (FAI and CsI), along with PbI_2_ (*X* mmol) and PbBr_2_ (1 − *X* mmol), in 1 ml of a dimethylformamide (DMF):dimethylsulfoxide (DMSO) (4:1) solvent mixture, which was then stirred continuously at 70°C overnight.

The spin-coating process consisted of two sequential steps at different rotation speeds – typically 1000 rpm for 10 s, followed by 5000 rpm for 30 s. To influence the surface morphology and microstructure, an antisolvent (chloro­benzene, typically 150 µl) was dropped onto the spinning substrate 15 s before the end of the second step. After deposition, the films were annealed at 100°C and then placed on an aluminium-foil-covered Petri dish and allowed to cool to room temperature for a few minutes. All fabrication steps were conducted inside a nitro­gen-filled glovebox to prevent exposure to moisture and oxygen. The sample preparation is more closely described by Ridzoňová *et al.* (2022 ▸), Horynova *et al.* (2025 ▸) and Peter Amalathas *et al.* (2025*b* ▸)

### 3.2. X-ray diffraction

XRD measurements were performed using a Rigaku SmartLab diffractometer equipped with a rotating copper anode in parallel beam geometry with a fixed incidence angle of 1°. The angular resolution of 0.5° in the diffraction plane was defined by a parallel-plate analyser in front of the detector. The axial divergence and acceptance were limited to 5° by Soller slits on the primary and secondary sides, respectively. The parallel beam geometry is suitable for this measurement because the low angle of incidence enhances the signal from the layer compared with the substrate, and the fixed angle of incidence ensures irradiation of the full surface area of the sample. The sample was kept in a polyethyl ether ketone dome in an argon atmosphere to eliminate potential decomposition of the sample caused by exposure to air and humidity. Outside of the well defined illumination intervals, the sample was kept in the dark at all times. For the measurement, we chose a 2θ interval that contains diffraction peaks 100, 110, 111, 200, 210, 211, 220 and 221. We chose the measurement time for each point to be 0.6 s, which, in combination with the scanning line detector mode, results in each measurement taking 70 min. This gives us a reasonable quality of the data and, at the same time, gives us a good enough time resolution to observe the relaxation of segregation in our sample. The measurements were conducted in a sequential manner, whereby each subsequent scan started immediately upon completion of the preceding scan.

### 3.3. Sample illumination

Illumination of the sample was done by a PhotoFluor II solar simulator by 89 North. The power of the solar simulator and distance to the sample were set such that the illumination of the sample was equivalent to an illumination by 1 Sun as calibrated by the photocurrent on a silicon detector. During the illumination we did not perform any diffraction measurements.

During illumination, the sample is heated by irradiation; however, it rapidly cools back to room temperature in the dark. Therefore, we expect to see no diffraction-peak shift due to the thermal expansion.

## 4. Results

Before the first illumination, we performed a measurement on a pristine sample. This measurement confirmed a cubic perovskite structure with lattice parameter *a* = 6.17 Å, and no macroscopic residual strain was observed. Then we illuminated the sample for 10 min and, immediately after illumination, we started a series of measurements to investigate the relaxation of photoinduced changes. After 53 h of relaxation, we illuminated the sample once more, this time for 30 min, and measured the relaxation again.

For a comparison of the simulated and measured data, we had to take into account several additional instrumental effects. First, the radiation passing through the X-ray mirror is not strictly monochromatic but contains two characteristic wavelengths, Cu *K*α_1_ (λ = 1.5406 Å) and Cu *K*α_2_ (λ = 1.5444 Å). This is implemented by adding a scaled copy of the peak to the appropriate 2θ position, according to the known wavelength separation and intensity ratio. The calculated diffraction curve was then convoluted with the instrumental resolution function obtained from the measurement of an LaB_6_ standard. Finally, a linear background was added separately for each peak.

In the final model, the fitted parameters are the r.m.s. deviation of the concentration σ, the correlation length of the concentration ξ, the radius of the grains *R*, the factor of asymmetry α and the lattice parameter *a*. Additionally, for each maximum, a scale parameter was added to account for the difference between the measured and predicted intensities of individual peaks. These scale parameters, including for example the texture and geometric influence of the diffractometer, do not change for subsequent measurements. All peaks of the given diffraction pattern were fitted together with the same parameters.

**Figure 5:**
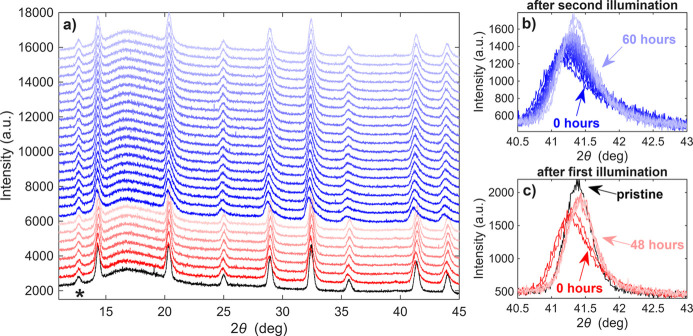
(*a*) Measured diffraction patterns before illumination (black), after 10 min of illumination (red) and after another 30 min of illumination (blue). The data are shifted vertically for visibility. Panels (*b*) and (*c*) show details of diffraction maximum 220 after the first and second illuminations. The diffraction profiles of individual maxima after illumination are broadened and asymmetrical, and this effect gradually relaxes when the sample is kept in the dark. The peak denoted by an asterisk belongs to PbI_2_ impurity.

In Fig. 5 ▸ we present all diffraction patterns measured before and after illumination. Before the illumination of the sample, we observe symmetrical diffraction maxima. After the short 10 min illumination, the peak shifts slightly to lower 2θ and decreases in intensity, but the most significant change is the new asymmetrical broadening of the peaks. All peaks broaden more in the direction of higher 2θ and peaks at higher diffraction angles have a greater broadening. Over time, these changes relax and after 48 h the peak shapes and positions are almost back to the original values. After this, the sample was illuminated once again, this time for 30 min. We observe similar behaviour as seen after the first illumination. During the entire procedure, we do not observe the creation or annihilation of any diffraction maxima. In addition to the expected diffraction maxima of the phase FA_0.83_Cs_0.17_Pb(I_0.6_Br_0.4_)_3_, we also observe a peak at 2θ = 12.7°. A single peak is insufficient for definitive phase identification; however, its position is consistent with the 001 reflection of hexagonal PbI_2_ (space group 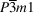), which is a common impurity phase observed in MHPs (Macpherson *et al.*, 2022 ▸). This peak does not change its intensity, position or shape during illumination/relaxation, indicating that this impurity remains stable under our conditions.

**Figure 6:**
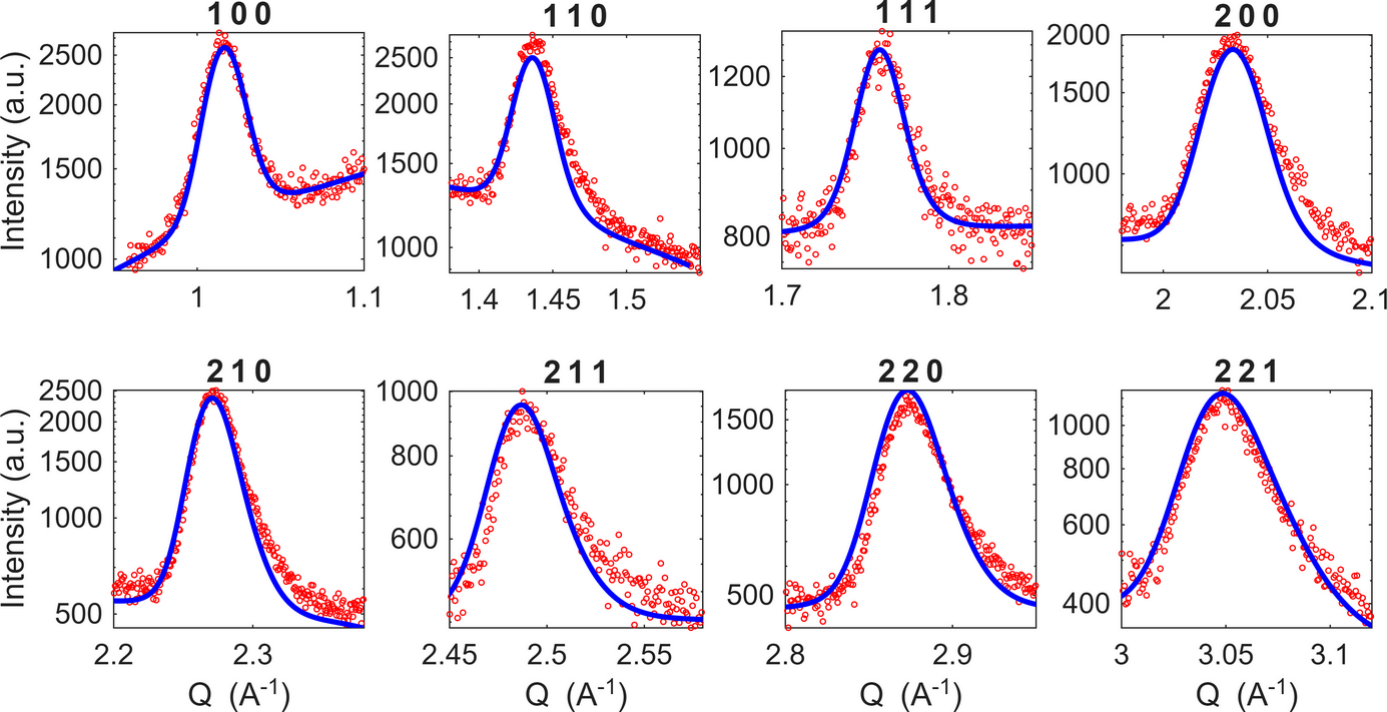
Diffraction maxima measured after 10 min of illumination (red) and fits by our model (blue). The *y*-axis scale is logarithmic. The parameters obtained from the fitting are σ = 0.13 ± 0.01, ξ ≥ 15 nm, *R* = 50 nm and α ≥ 5.

**Figure 7:**
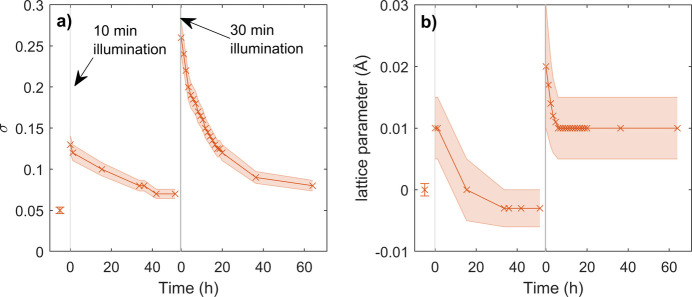
Evolution of (*a*) r.m.s. deviation of concentration and (*b*) lattice parameter during relaxation of segregation. The shaded areas represent error bars and grey areas mark the time of illumination of the sample. The *x* axes show the time since the last illumination (relaxation time).

We fitted all diffraction patterns by our model. In Fig. 6 ▸ we present a comparison of the measured data and the fit for each diffraction pattern measured after the first illumination; for other fitted XRD patterns see Fig. S1 in the supporting information. For comparison of the simulated and measured data, we recalculated the 2θ angle to reciprocal-space vector *Q*. Our model predicts well all the described effects. Root-mean-square deviations of concentration σ for all measurements, shown in Fig. 7 ▸(*a*), increase during illumination and exponentially relax in the dark. The mean radius of the crystalline grains is 50 nm for all measurements. This crystallite size is in good agreement with the values determined by scanning electron microscopy (Fig. S2). The correlation length in all measurements is higher than the limit of sensitivity of the simulation, which is 15 nm. We assume the correlation length to be the same as the crystallite size. Finally, for all measurements we observe positive asymmetry factors of values higher than 5, which is the sensitivity limit of the model to this parameter. This means that there are bromine-rich inclusions in a given volume that have a slightly higher iodine concentration than the uniform concentration. This asymmetrical fluctuation of concentration is the only possible distribution of concentration that can explain the asymmetrical broadening of the diffraction maxima that is *hkl* independent and in the direction of higher *Q*. Within the presented model, an asymmetric concentration distribution (α > 1) is required to reproduce an *hkl*-independent asymmetric broadening towards higher *Q*, whereas symmetric fluctuations (α = 1) always produce an *hkl*-dependent asymmetry direction.

While the majority of the observed shift of the maxima can be explained by our model [Figs. 4 ▸(*e*)–4 ▸(*h*)], we do observe a small change in the lattice parameter [as shown in Fig. 7 ▸(*b*)] that cannot be explained by our model. It also cannot be attributed to temperature changes, as we expect that thermalization has finished before the measurement of the first peak. This is confirmed by the observation of no changes in the lattice parameters for different maxima during the initial measurement following illumination.

For an explanation of this additional change in the lattice parameter we will consider a spherical highly Br-rich inclusion in a slightly I-rich volume, which serves as a simplification of the distribution obtained by the fitting. The inclusion is expected to exhibit a lattice parameter smaller than that of the surrounding matrix and, given the requirement for the conservation of numbers of unit cells, the inclusion will try to contract compared with its original volume with the mean Br concentration. This will lead to strain in both the inclusion and the surrounding volume. This strain can be calculated using the theory developed by Eshelby (1957 ▸), and the mean lattice parameter of such a system is larger than the lattice parameter of a perovskite with the mean Br concentration. Additionally, the lattice parameter may be further affected by the incorporation of Br or I ions at grain boundaries, within lattice defects, or in other regions that do not contribute to coherent diffraction. This changes the mean Br concentration within coherently diffracting domains and therefore influences the mean lattice parameter.

## 5. Discussion

Using the presented method, we were successfully able to determine the distribution of halide ions within an MHP during illumination and relaxation. This method is a complementary approach to photoluminescence spectroscopy (PL), which probes primarily the band gap of the volume with the lowest number of defects in the MHP material and, due to thermodynamics, does not see the Br-rich region. XRD, on the other hand, investigates crystal structure and real structure in the whole material volume equally.

In this work we have not considered strain relaxation at surfaces, defects and grain boundaries. Strain relaxation will lead to a smaller volume-force density in equation (12) and therefore to a smaller local displacement. This implies a limitation of our model, particularly in systems characterized by a high density of strain relaxation centres, such as grain boundaries. In such contexts, the model tends to underestimate the r.m.s. deviation of the Br concentration. This can be accounted for by adding a variable that describes the relaxation into equation (13) in Appendix *A*[App appa]. The question remains how to quantify the relaxation at different types of relaxation centres. Recently, it was shown by theoretical calculations using the Cahn–Hilliard equation that strain relaxation is in fact needed for halide demixing (Holý *et al.*, 2025 ▸).

We observed the creation of small Br-rich regions and larger slightly I-rich regions during illumination, and subsequent slow and imperfect relaxation in the dark on a timescale of hours. A similar concentration distribution could also be represented by spherical inclusions of high concentration in a volume of slightly low concentration. However, to describe such an arrangement, we need many more parameters, which makes the fitting cumbersome and ambiguous. Our efforts to describe the measured data by various alterations of spherical inclusions yielded significantly worse fits than the random concentration model.

Although most published studies agree on the timescale of the processes, not a single study explicitly reports the creation of highly Br-rich perovskite regions in a slightly I-rich volume. For studies using only PL as a probe of local halide concentration, this is logical, as the I-rich regions have higher PL activity [because the bandgap of FAPbI_3_ (Tang *et al.*, 2020 ▸) is lower than that of FAPbBr_3_ (Zhang *et al.*, 2018 ▸)] and therefore dominate the spectra. By means of high-resolution trans­mission electron microscopy, Funk *et al.* (2023 ▸) revealed the creation of sub-5 nm Br-rich inclusions in CsPb(Br_*x*_I_1−*x*_)_3_. However, due to the structural similarity of CsPbBr_3_ and PbBr_2_, they were unable to distinguish decisively between the phases and, on the basis of previously published research, attributed these regions to the PbBr_2_ phase. In the few published papers using XRD to investigate halide segregation in FA_*y*_Cs_1−*y*_Pb(Br_*x*_I_1−*x*_)_3_ (Knight *et al.*, 2021 ▸; Sutter-Fella *et al.*, 2018 ▸) we do not see any clear agreement on halide distribution in illuminated samples. For the case of MAPb(I_*x*_Br_1−*x*_)_3_ perovskites, although not directly stated (Duong *et al.*, 2017 ▸), a splitting of the diffraction peaks is observed that is consistent with the creation of small Br-rich regions within a slightly I-rich volume.

While the difference between I^−^ and Br^−^ migration activation energies in FA_*y*_Cs_1−*y*_Pb(Br_*x*_I_1−*x*_)_3_ is smaller than in MA-based perovskites (Oranskaia *et al.*, 2018 ▸), the migration barrier for I^−^ is still lower than that for Br^−^. This means that the observed creation of highly Br-rich regions in a low I-rich volume suggests that the I^−^ ions have migrated outwards from some nucleation site such as a grain boundary, interface or lattice defect.

## 6. Conclusions

In this work, we have developed and validated a quantitative XRD method capable of resolving light-induced halide segregation in mixed-halide perovskites. By combining elastic strain field calculations with a diffuse scattering model, we were able to reconstruct the spatial distribution of Br and I ions within FA_0.83_Cs_0.17_Pb(I_0.6_Br_0.4_)_3_ thin films after illumination and subsequent relaxation in the dark. The model successfully reproduces illumination-induced broadening, asymmetry and shifts of diffraction maxima, and enables the determination of the r.m.s. fluctuation, asymmetry and correlation length of the halide concentration.

Our analysis reveals that illumination leads to the formation of highly Br-rich regions embedded within a slightly I-rich matrix, followed by slow incomplete relaxation over hours in the dark. These findings provide direct structural evidence for asymmetric halide redistribution.

The presented approach offers a robust complementary alternative to photoluminescence-based techniques, enabling bulk-sensitive and composition-specific insight into the mechanisms of light-induced demixing in metal halide perovskites.

## Supplementary Material

All XRD data and their fits, SEM images. DOI: 10.1107/S1600576726002475/xx5095sup1.pdf

## Data Availability

The data supporting the findings of this study are available from the corresponding author upon request.

## Appendix A Strain field calculation

We assume that the MHP crystal is cubic and that its lattice parameter depends linearly on the local Br concentration *c*_Br_(**r**),

where *a*_0,1_ are the MHP lattice parameters for *c*_Br_ = 0, 1, respectively.

The local displacement **u**(**r**) in an infinite crystal obeys the equilibrium equation

where *C*_*jkmn*_ are the elastic constants of the MHP in four-index notation (assumed not dependent on *c*_Br_) and **f**(**r**) is the volume-force density. In cubic crystals this can be expressed using the gradient of *c*_Br_,

Here we use the two-index notation of the elastic constants *C*_11_ ≡ *C*_*xxxx*_, *C*_12_ ≡ *C*_*xxyy*_.

In an infinite crystal, equations (13) and (14) can be solved by the Fourier method, putting

which converts the system of partial differential equations (13) to a set of linear algebraic equations that can be solved directly:

Here  is the elastic Green function defined as

In all these formulas, the superscript (FT) denotes Fourier transformation.

## Appendix B Symmetry of diffuse diffracted intensity from strain component

For the simple case of a spherical inclusion or an inclusion with a Gaussian profile of concentration, we can always choose the coordinate system such that the centre of the sphere is the origin of the coordinate system. From spherical symmetry it is immediately clear that for any strain field **u**(**r**) created by this inclusion, **u**(−**r**) = −**u**(**r**) must be true. If in equation (11) we consider only the strain component, we can express the diffuse part of the diffracted intensity with only the strain component from equation (6) as

Therefore, from the symmetry condition **u**(−**r**) = −**u**(**r**),  =  follows, and therefore the absolute value of  and the diffracted intensity are both symmetrical.

A similar symmetry condition can be found for a random distribution of concentration with a correlation function given in the main text by equation (7) assuming an asymmetry factor α = 1. We express the diffuse part of the diffracted intensity with only the strain component as

Since **u**(**r**) is a random variable with a Gaussian distribution and zero mean, we can rewrite the average as

and

Substituting this into equation (19) we find that again , *i.e.* the absolute value of  and the diffracted intensity are both symmetrical, even for a random distribution of concentration with α = 1. As explained in the main text, the asymmetry of the intensity distribution visible in Figs. 4 ▸(*a*)–4 ▸(*d*) is caused solely by the interference of the strain term 〈*S*_**h**_〉{exp[−*i***h** · **u**(**r**)] − 1} with the contribution of the chemical contrast Δ*S*_**h**_(**r**)exp[−*i***h** · **u**(**r**)].
