# Supplementary material for: Quantitative analysis of light-induced ion segregation in mixed-halide perovskites
Source: J Appl Crystallogr. 2026 May 20;59(Pt 3):879–88. doi: 10.1107/S1600576726002475 (PMC13224788; doi:10.1107/S1600576726002475)
Supplement: Supplementary file 1 [file j-59-00879-sup1.pdf]

JOURNAL OF APPLIED CRYSTALLOGRAPHY

Volume 59 (2026)

Supporting information for article:

Quantitative analysis of light-induced ion segregation in mixed-halide perovskites

Petr Machovec, Lukáš Horák, Milan Dopita, Neda Neykova, Lucie Landová, Jakub Holovský and Václav Holý

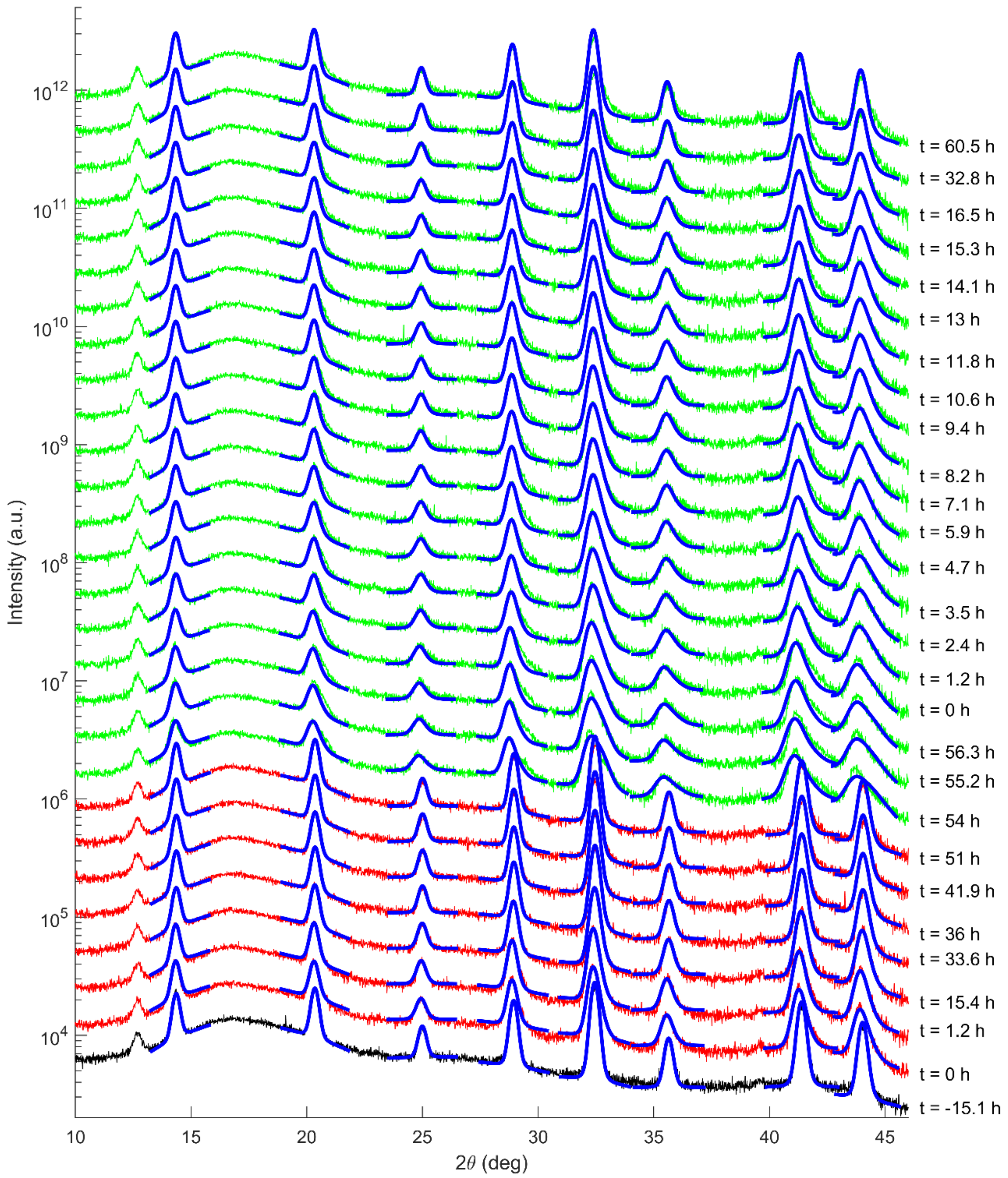

Figure S1 All measured XRD patterns and their fits. The data are proportionally vertically shifted based on the time when they were measured. The black pattern is measured before the first light soaking, the red patterns were measured after 10 minutes of light soaking, and the green patterns were measured after another 30 minutes of light soaking. All fits are shown in blue. The time of the measurement since the last illumination is shown next to each diffraction pattern.

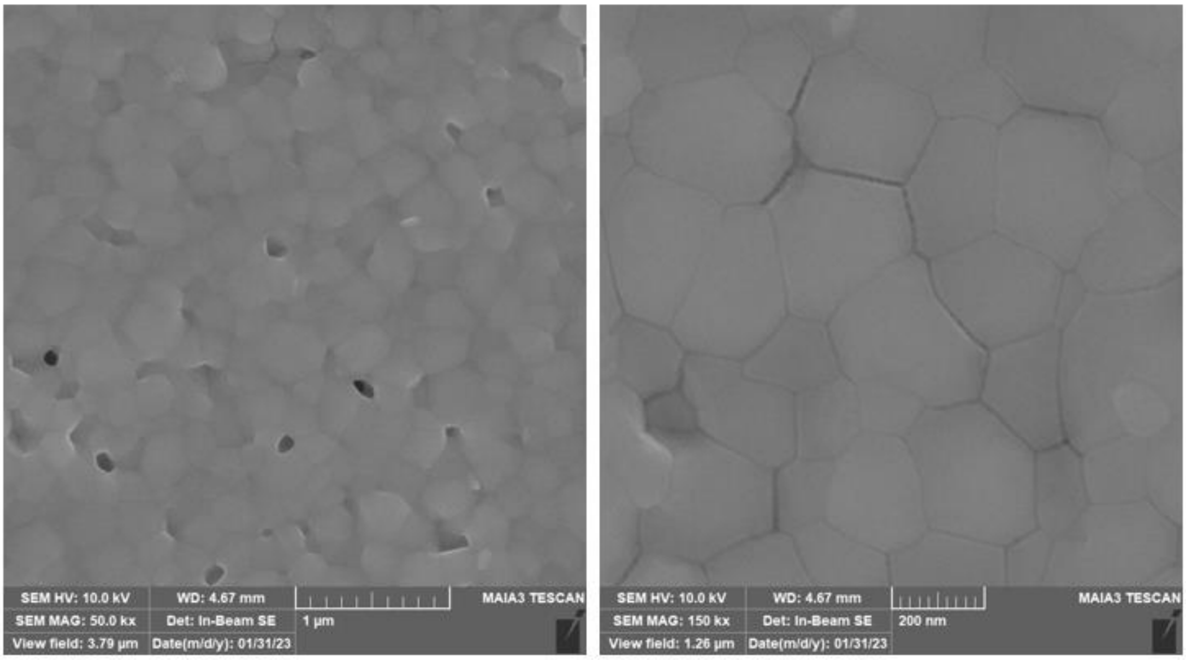

Figure S2 SEM secondary electrons images of the sample surface. The SEM measurement reveals a grain radius ranging from 50 to 100 nm. The layer exhibits some small voids, but is otherwise homogeneous.
